# A graphene oxide/polyaniline nanocomposite biosensor: synthesis, characterization, and electrochemical detection of bilirubin†

**DOI:** 10.1039/d3ra06815c

**Published:** 2023-12-12

**Authors:** Noor sabah Ahmed, Chou-Yi Hsu, Zaid H. Mahmoud, Hamidreza Sayadi, Ehsan kianfar

**Affiliations:** a College of Sciences, University of Diyala Iraq; b Department of Pharmacy, Chia Nan University of Pharmacy and Science Tainan Taiwan; c Chemistry Department, College of Science, University of Diyala Iraq zaidhameed_91@yahoo.com; d Department of Chemical Engineering, Faculty Shahrood Branch, Shahrood Branch Shahrood Iran; e Department of Chemical Engineering, Arak Branch, Islamic Azad University Arak Iran ehsan_kianfar2010@yahoo.com; f Young Researchers and Elite Club, Gachsaran Branch, Islamic Azad University Gachsaran Iran ehsankianfar775@gmail.com

## Abstract

The level of free bilirubin is a considerable index for the characterization of jaundice-related diseases. Herein, a biosensor was fabricated *via* the immobilization of bilirubin oxidase (BOx) on graphene oxide (GO) and polyaniline (PANI) that were electrochemically co-precipitated on indium tin oxide (ITO) conductive glass. The structural enzyme electrode was characterized by FTIR, XRD, and Raman spectroscopy, while the spectral and thermal properties were investigated by UV-vis and thermogravimetric analysis (TGA). Owing to the activity of the fabricated BOx/GO@PANI/ITO biosensor, it could detect free bilirubin with good selectivity and sensitivity in a low response time. The electrochemical response was studied using electrochemical impedance spectroscopy (EIS) and cyclic voltammetry (CV). At polarization potential 0.2 V *vs.* Ag/AgCl, the fabricated sensor illustrated a response in only 2 s at 30 °C and pH 7.5. The LOD and LOQ for the BOx/GO@PANI/ITO biosensor were calculated and found to be 0.15 nM and 2.8 nM, respectively. The electrochemical signal showed a linear response in the concentration range 0.01–250 μM. At 5 °C, the biosensor demonstrated a half-time of 120 days, through which it could be utilized 100 times at this temperature conditions. By using a common colorimetric method, the data on bilirubin levels in serum showed a determination coefficient (*R*^2^) of 0.97.

## Abstract 2

The level of free bilirubin is a considerable index for the characterization of jaundice-related diseases.

## Graphical abstract

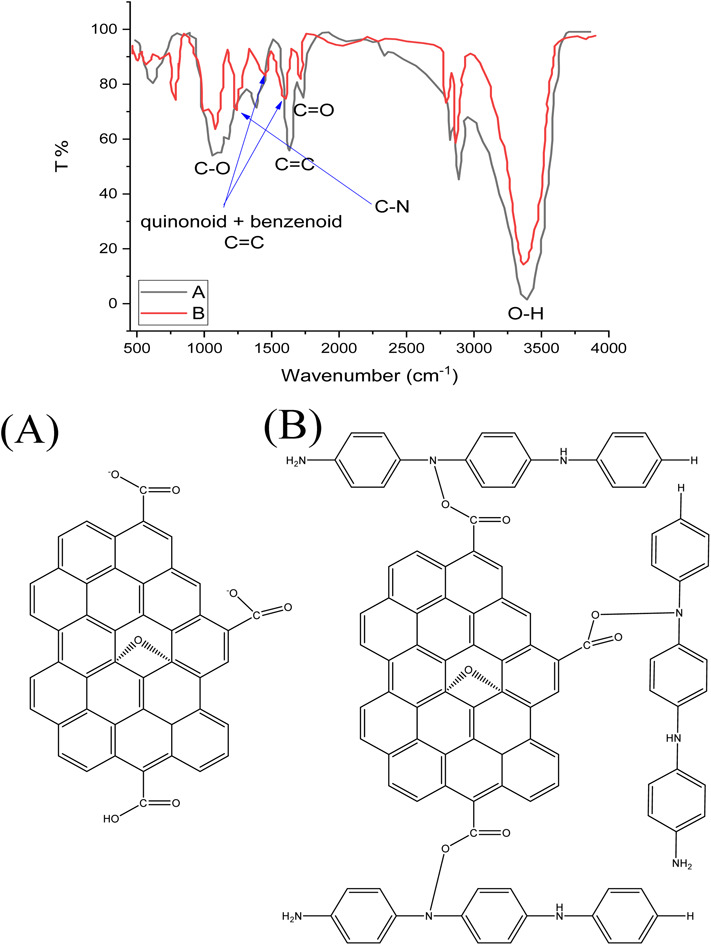

## 1. Introduction

Most of the bilirubin synthesized comes from the metabolic breakdown of hemoglobin, and it is classified into two kinds: direct bilirubin and free bilirubin.^1–3^ The latter is a substantial index for determining the toxicity of bilirubin and an concentration above 50 μM may cause hemolysis, hepatitis, or jaundice-related cirrhosis.^4–7^ Moreover, newborn livers, particularly those of preterm babies, are not developed enough to remove or get rid of free bilirubin, and therefore neonatal jaundice is considerably common. The high concentration of free bilirubin can cause brain damage risk in newborns or even death.^8–11^ Advanced instrumentation with a high precision, fast response, low-cost, and ease of operation is required for studying the biological and characteristic importance of free bilirubin.^12–14^ Spectroscopic measurements using a diazo reaction is the most commonly utilized free-bilirubin quantification method but requires the pretreatment of samples and long reaction times.^15–19^ The fluorescence analysis method is selective and highly sensitive toward the free type,^20–23^ but huge and expensive spectral tools and trained operators are needed. Electrochemical sensors have emerged as powerful and potential tools for quantifying free bilirubin, and because of their low cost, ease of utilization, and small sizes, they are potential candidates for medical applications.^24–27^ For example, electrochemical sensors have provided quick and simple methods for the detection of free bilirubin by utilizing bilirubin oxidase,^28–31^ which coverts bilirubin into biliverdin. However, biosensors have the disadvantages of poor stability and high cost.^32–35^ To overcome these obstacles, non-enzymatic biosensors utilizing nanomaterials have gained much interest because of their high activity, stability, and low cost. For example, Au and Ag nanoparticles^36–39^ have been reported as substituents for BOx in the free-type catalytical oxidation for biosensing. However, the construction of gold and silver non-enzymatic catalysts with high activity prepared *via* complicated steps as an improvement over inefficient, simple free-bilirubin biosensors is still challenging.^40–42^ The utilization of nanomaterials to enhance electrochemical sensor performance has been very common in recent years. Among the various nanomaterials, graphene and its derivatives have emerged as interesting materials owing to their high electrical conductivities and relatively low cost of preparation.^43–45^ Graphene has a large surface area, but the interactions between graphene sheets cause an aggregation which efficiently decreases its surface area. To overcome this issue, doping graphene with other nano oxides is proposed.^46–48^ Graphene is composed of carbon atom rings with sp^2^ hybridization and active oxygen groups. GO makes the surface of graphene active leading to the association of functional groups.^49–52^ The prompt electron transport occurs at the surface of the edge planes when contrasted to the essential planes for the biosensors prepared with graphene-dependent materials.^53–55^ These defects in the modified graphene can be exploited for biosensor applications.^56–59^ Polyaniline (PANI) is generally utilized as a conducting polymer in biosensors due to its inveterate stability in atmospheric conditions.^60–62^ Polyaniline has a large surface area for congealing enzymes or nanoparticles because of its porous structure. In addition, it is a good material for biosensor applications due to its stability and excellent electric conductivity.^63–66^ To prevent agglomeration or aggregation, GO was doped into the PANI to increase the interaction between GO and PANI materials. A nanocomposite of GO nanosheets with PANI (GO@PANI) is anticipated to show higher conductivity compared to the individual components to improve the response of biosensors in terms of stability, sensitivity, and electric conductivity. In this work, we promote a new strategy for immobilizing BOx on the GO@PANI modified indium tin oxide (ITO) coated glass plate electrode. Moreover, optimization and characterization of the prepared electrodes and their application for determining bilirubin in the blood serum were also performed.

## 2. Experimental

### 2.1 Materials

All materials used in preparing the active electrodes were utilized without any future purification. BOx (15 IU mg^−1^), tris hydrochloric acid, bilirubin, and glutaraldehyde were supplied by Sigma Aldrich Co. Sodium nitrate, 4-amino phenzophenone potassium, sodium sulphate ferrocyanide, phenol, and horseradish were purchased from Fluka Co. Ammonium persulfate, graphite, potassium permanganate, hydrogen peroxide, and aniline were procured from Merck Co. Indium tin oxide (ITO) conductive glass with transmission 85% and resistance approximately 7 Ohm sq^−1^ was supplied by Sigma Aldrich.^67–69^

### 2.2 Assay of free BOx

The examination depends on measuring hydrogen peroxide (H_2_O_2_) that is released as bilirubin is oxidized by BOx.^34,70,71^ For 15 min and 37 °C, (0.8 ml, 0.25 M, pH = 8.5) of tris hydrochloric acid, (0.1 ml, 34 μm) bilirubin solution, and (0.1 ml, 5 U ml^−1^) of BOx were mixed together. Then, (1 ml, 0.45 M, pH = 7.0) of sodium phosphate, containing 40 mm 4-aminophenazone, 10 mg horseradish, and 1000 mg phenol were added to the mixture and kept incubated for 15 min at 37 °C. Finally, the absorption of the reaction mixture solution was read at 520 nm and the H_2_O_2_ concentration was extrapolated from the standard curve.

### 2.3 Synthesis of GO and GO-PANI nanocomposite

Graphite was converted to stable graphene oxide (GO) nanosheets by following the modified Hummers' method reported in our recent work.^35,72,73^ The polyaniline (PANI) chains were doped on the GO nanosheets during the *in situ* oxidative polymerization using ammonium persulphate as an initiator. For this purpose, (10 ml, 2 M) of HCl containing 3 ml of aniline solution was prepared under ice conditions, and 30 ml of aqueous GO solution (10 g/500 ml) was added to the solution. Then, the mixture was stirred for 30 min in an ice bath for 30 min. After that, (20 ml, 1 M) ammonium persulphate was dripped into the mixture until a black-green precipitate was formed, which confirmed the formation of PANI. Finally, the formed precipitate was isolated and washed 4 times with distilled water and dried at 80 °C for 2 h.

### 2.4 Electrodeposition of GO-PANI onto ITO conductive glass

By using cyclic voltammetry, GO-PANI was placed onto the ITO substrate (50 mm × 50 mm *×* 1.2 mm). Firstly, the ITO substrate was immersed in a solution of 25 mg of GO, (15 mM, 3 ml) aniline dispersed in 5 ml of 50 mM NaClO_4_. Then, the potential range of −0.2 to +0.8 V was applied for 15 polymerization cycles with scanning of 100 mV s^−1^. The cycle number affects the production of the nanocomposite film on the electrode surface.

### 2.5 Preparation of enzyme electrode (BOx/GO@PANI/ITO electrode)

A mixture of glutaraldehyde (GA), BSA, and BOx was immobilized on the surface of the GO@PANI/ITO electrode to fabricate an enzyme electrode. To prepare 15 μL of the mixture, 5 mL of glutaraldehyde (1.25% v/v in distilled water) was added to the solution containing 0.4 mg BSA and 0.1 BOx in (10 μL, pH 7, 0.1 M) the phosphate buffer solution. 5 μL of the crosslinked solution of BSA and BOx was dropped on the GO@PANI/ITO electrode surface and dried at 30 °C. Finally, after 24 h, the prepared electrode was washed with 5 ml of the phosphate buffer solution to remove the unbound enzyme.^74–76^

### 2.6 Response measurement and optimization factors of the BOx/GO@PANI/ITO electrode

Cyclic voltammetry measurements were carried out utilizing three electrodes with three mixture solutions as an electrolyte containing (10 ml, 0.1 M) KCl, (5 ml, 0.1 M, pH 7.5) sodium phosphate buffer, and (0.1 ml, 0.1 mM) of bilirubin with a potential range between −0.4 to 0.4 V. To determine the optimum concentration of the enzyme, the reaction was executed at different enzyme concentrations in the range of 100 to 500 IU. Many factors, such as pH impact, bilirubin concentration, temperature, and incubation time were studied to optimize the performance of the BOx/GO@PANI/ITO electrode. The optimum pH was obtained in the range 7–10, while the temperature was set in the range of (25 to 50 °C) in increments of 5 °C. For monitoring the response of the fabricated BOx/GO@PANI/ITO electrode at different bilirubin concentrations, the range of 0.01–250 μm was studied.

## 3. Results and discussion

### 3.1 Structural characterization

**Fig. 1:**
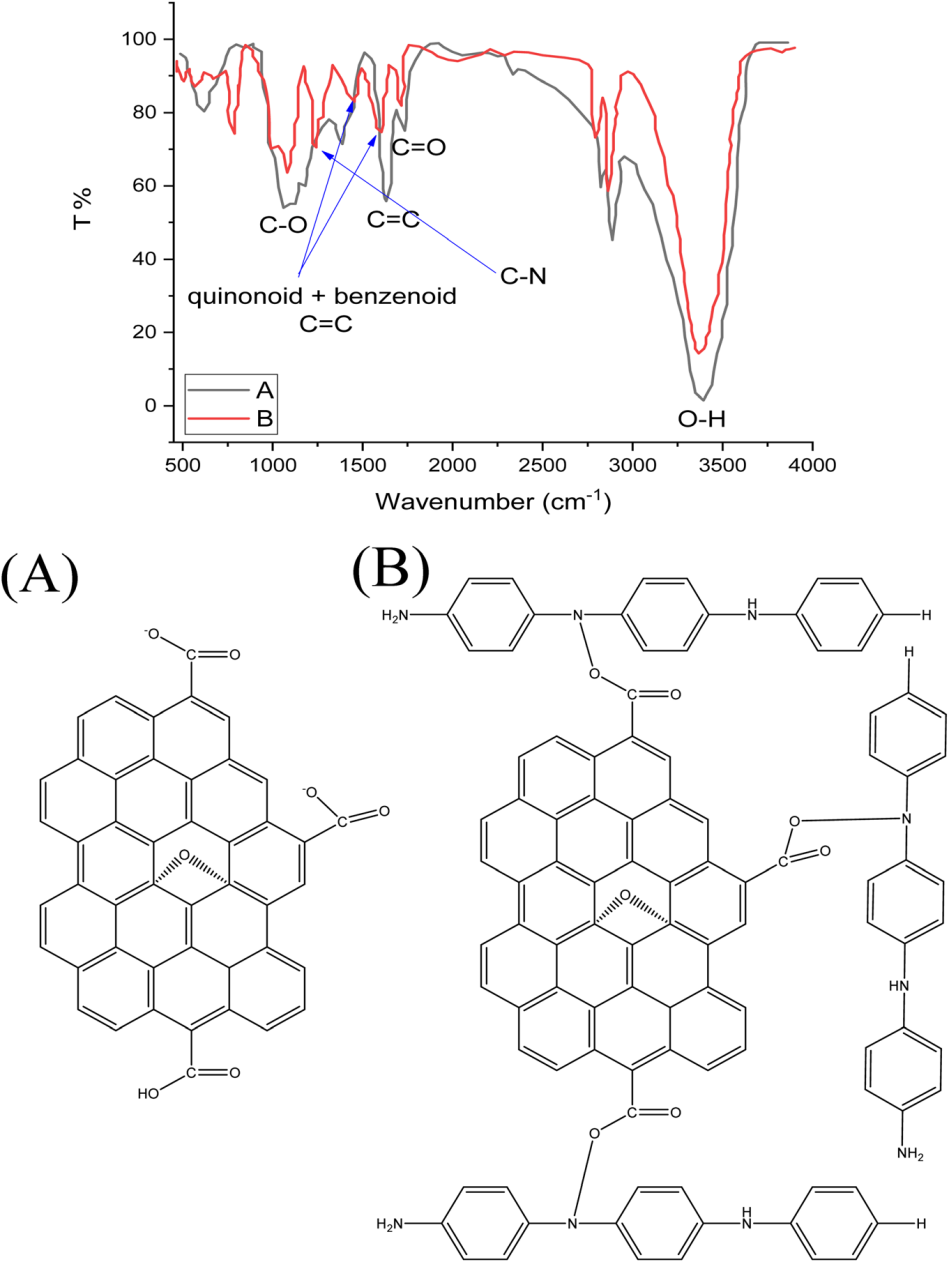
FTIR spectra of (A) GO and (B) GO@PANI.

FTIR spectra of GO and GO-PANI nanocomposite are shown in Fig. 1a and b, respectively. In Fig. 1a, two peaks centered at 1643 and 1735 cm^−1^ correspond to C=C and C=O stretching bands, respectively.^36,77–79^ A broad band located at 3420 cm^−1^ is assigned to O–H deformation in the COOH group. Moreover, a strong band located at 1118 cm^−1^ is due to the C–O in the epoxide group (C–O–C). The FTIR spectrum of PANI deposited on GO is illustrated in Fig. 1b. Two absorption peaks centered at 1474 and 1622 cm^−1^ correspond to quinonoid and benzenoid C=C groups, respectively. In addition, a new peak appearing at 1300 cm^−1^ is assigned to C–N due to the covalent bonding of PANI with GO sheets during the reaction with the epoxide ring.^37,80,81^

**Fig. 2:**
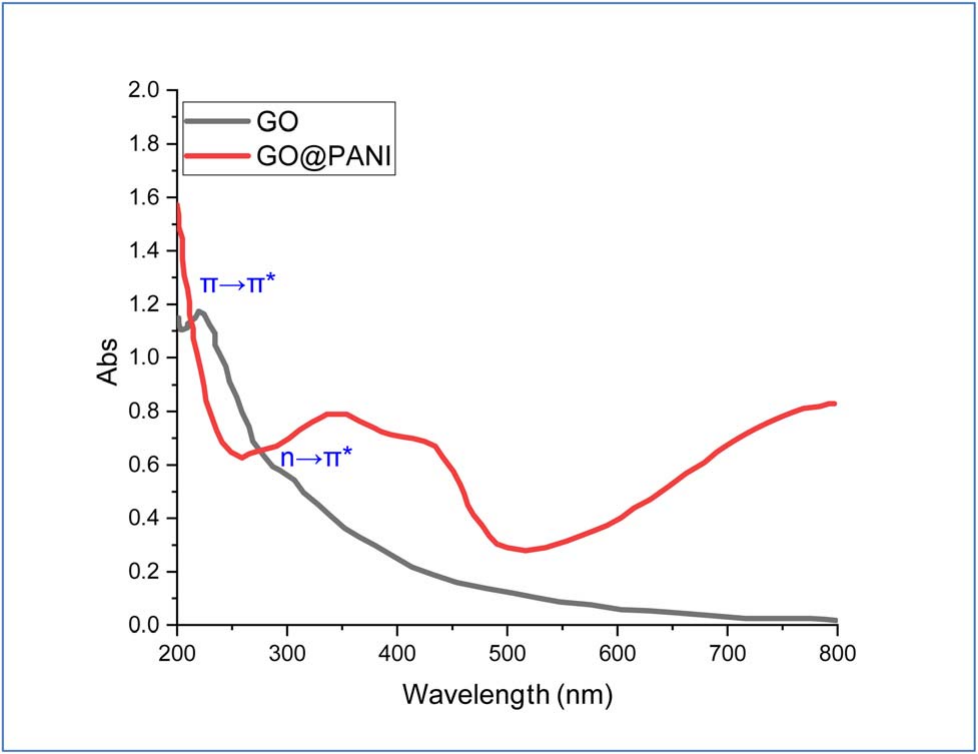
UV-vis spectra of GO and GO@PANI.

UV-vis spectra of GO and GO-PANI nanocomposite are depicted in Fig. 2. The GO spectrum shows two absorption bands located at 219 and 307 nm, which correspond to π → π* (C=C) and *n* → π* (C=O) groups, respectively.^38,82–84^ For the GO-PANI spectrum, the absorption band of π → π* almost disappeared after incorporating PANI, which indicated precipitation of PANI over GO sheets, causing weakening of the absorption of the benzene rings. Moreover, a new absorption band is shown centered at 300–500 nm, which is related to the binding of PANI with GO sheets.^39,85–87^

**Fig. 3:**
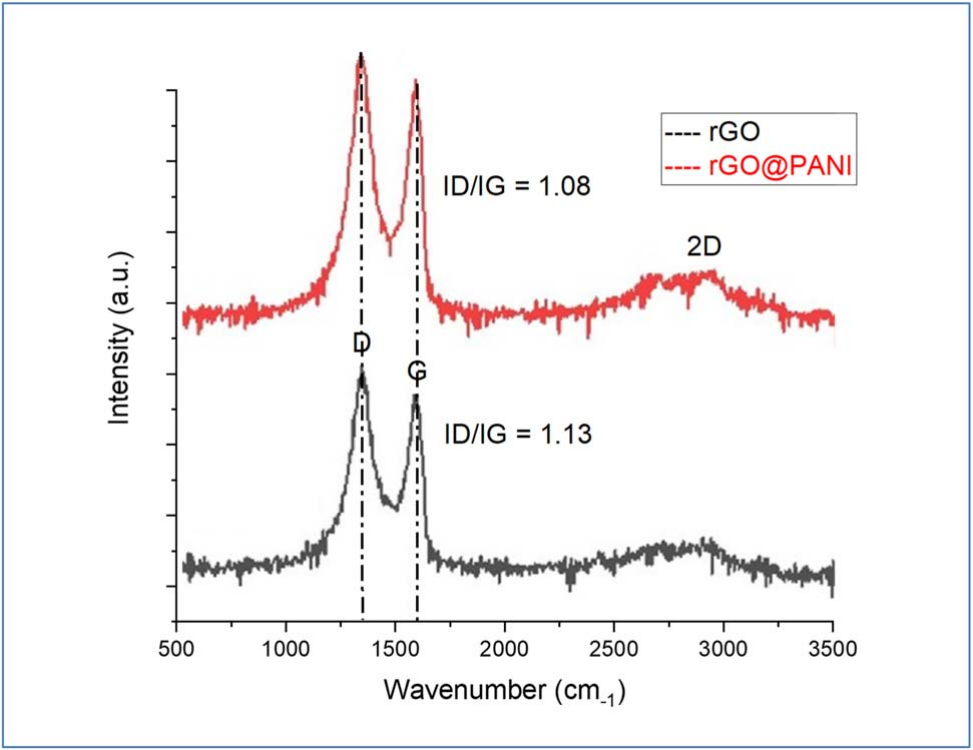
Raman spectra of GO and GO@PANI.

Raman spectra of GO and GO-PANI nanocomposite are demonstrated in Fig. 3. Two characteristic bands are associated with the defect density of GO sheets and sp^2^ graphitic carbon bonds, which are centered at 1350 and 1586 cm^−1^, called D and G-bands, respectively. For GO-PANI, the results show red shifting in the D band from 1350 to 1333 cm^−1^, which indicates the π → π* interaction of PANI with GO sheets.^40^ On the other hand, the results showed that the 2D band intensity was decreased after the precipitation of PANI over GO indicating the presence of more PANI layers than GO layers and the reduction of GO by PANI.

**Fig. 4:**
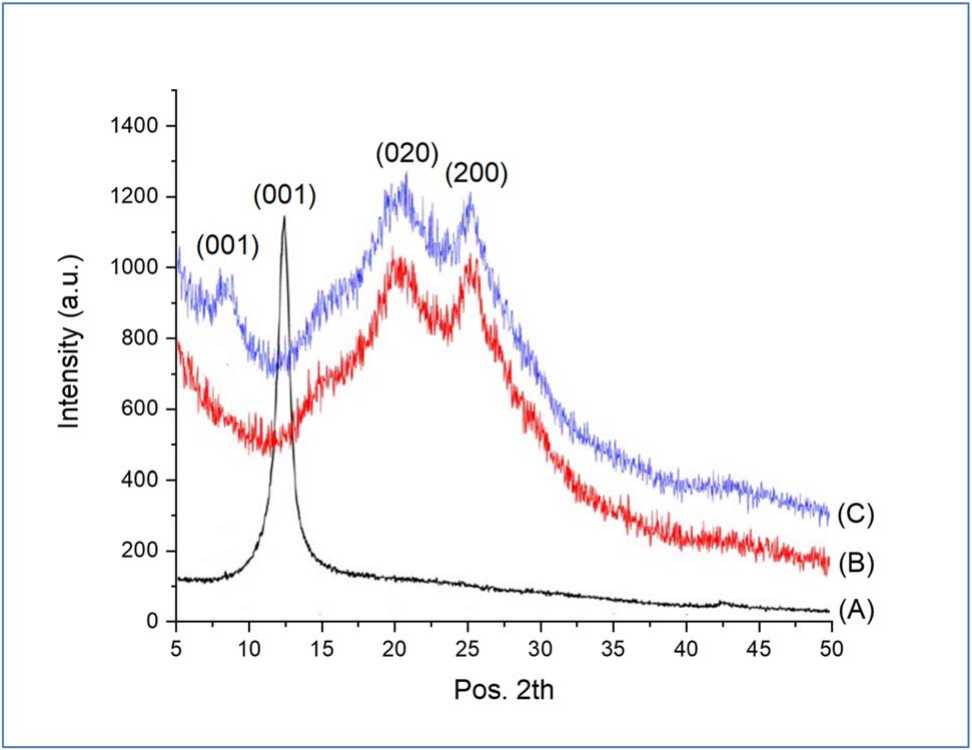
XRD patterns of (A) GO, (B) PANI, and (C) GO@PANI.

To confirm the structure of the prepared GO-PANI, XRD analysis of pure GO, PANI, and GO-PANI nanocomposite was performed and the results are shown in Fig. 4a–c. As shown in Fig. 4a, a strong and sharp diffraction peak centered at 2*θ* = 12.53° related to the interlayer spacing of 0.61 nm of pure GO was observed.^41^ The XRD pattern of pure PANI is shown in Fig. 4b. Two diffraction peaks located at 2*θ* = 19.9° and 25.20°, corresponding to (020) and (200) crystal planes of the emeraldine salt form were observed. For GO-PANI nanocomposite, it can be noted that the diffraction peak related to pure GO is shifted from 12.53 to 8.76 nm, which is assigned to the distance of 1.02 nm, as shown in Fig. 4c. The expansion in the distance of layer is due to the interaction of PANI between the sheets of GO.^42^

**Fig. 5:**
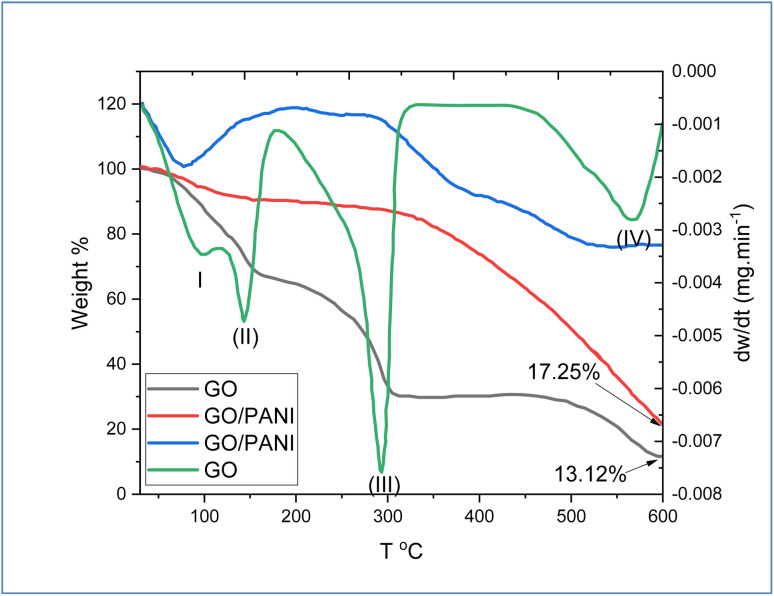
TGA and DTG of GO and GO@PANI.

To investigate the thermal stabilization and the impact of the incorporation of PANI chains into the GO sheets, TGA/DTG analysis was performed and the results are shown in Fig. 5. Derivative thermogravimetry (DTG) plot analysis of GO indicated four major steps for losing the mass at zones (1) 100–115 °C, (11) 115–170 °C, (III) 170–300 °C, and (IV) 400–550 °C, which are related to the removal of adsorbed water on GO surface, removal or decomposition of hydroxyl group, decomposition of epoxy and carboxylic acid functional group, and finally analysis or decomposition the skeleton carbon of GO, respectively.^43^ At 600 °C, the remaining weight was about 13.12% from GO. In the same Fig. 5, the results exhibited the TGA/DTG of GO modified by PANI. The results showed no clear loss of weight step up to 350 °C, which indicates an increase in the thermal stability of GO during the interaction of PANI with oxygenic functional groups of GO. Above 340 °C, the results show an increase in the weight loss of PANI-GO nanocomposite, which is due to the decomposition of PANI chains.^44^ Moreover, at 600 °C, the remaining weight of the nanocomposite was approximately 17.25%.

### 3.2 Construction of the bilirubin biosensor

**Fig. 6:**
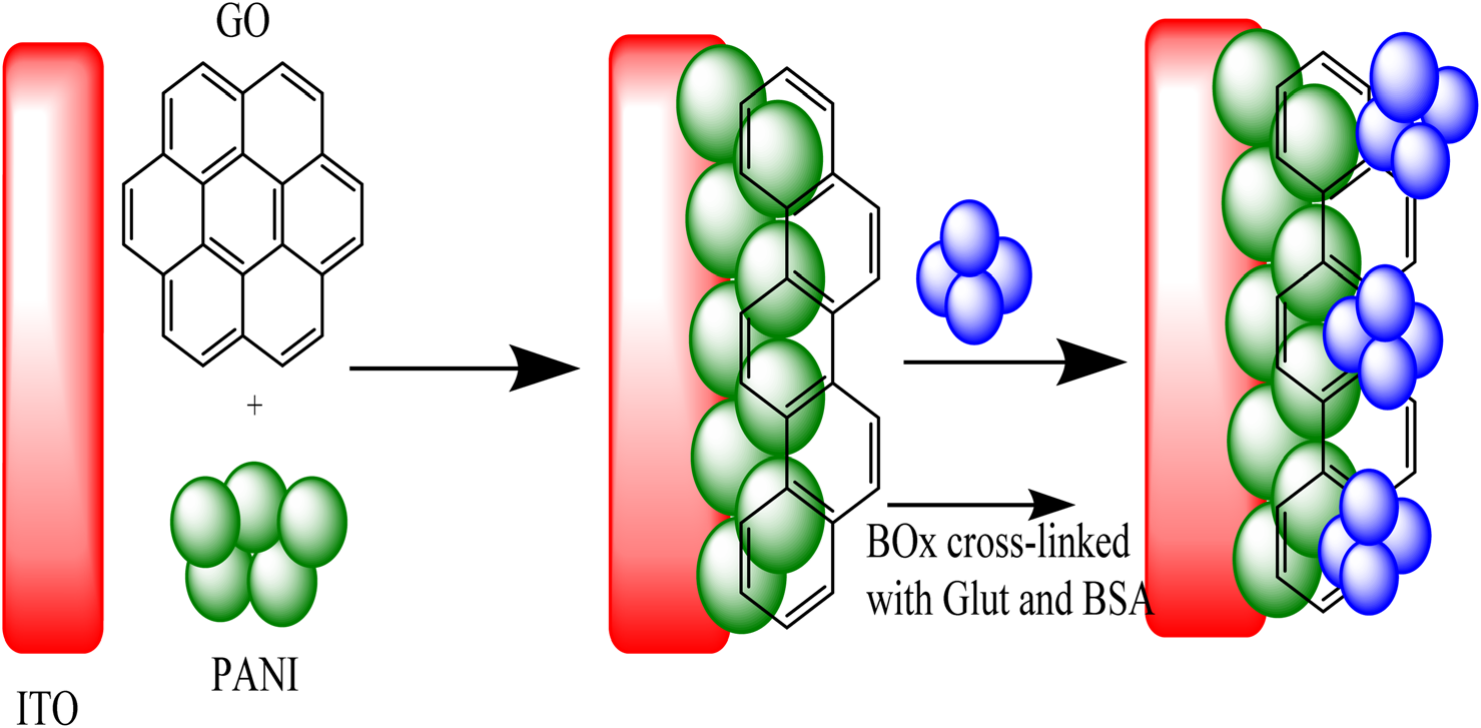
A schematic demonstration of the fabrication steps of the bilirubin biosensor.

The prepared enzyme electrode based on the immobilization of BOx on GO nanosheets/PANI nanocomposite modified ITO electrode is shown in Fig. 6. By using a simple electrochemical method, the mixture of GO and PANI was co-precipitated on the bare ITO conductive glass. After that, several modifications were performed on the GO@PANI/ITO electrode by adding an extensively crosslinked solution of BOx-BSA/GA on the surface of the electrode. The linking was performed by attaching the CHO group of GA with the NH_2_ group on the surface of the enzyme and attaching another CHO group with NH_2_ of BSA, thus attaching or crosslinking the product to a stable complex of BOx. The cyclic voltammetry (CV) indicated that the GO@PANI/ITO electrode promoted the currents compared with the PANI/ITO electrode, which indicated high surface area and more charge transfer pathway provided by the GO nanosheets, thus improving the response of the biosensor and boosting the sensitivity.

### 3.3 Electrochemical characterization

**Fig. 7:**
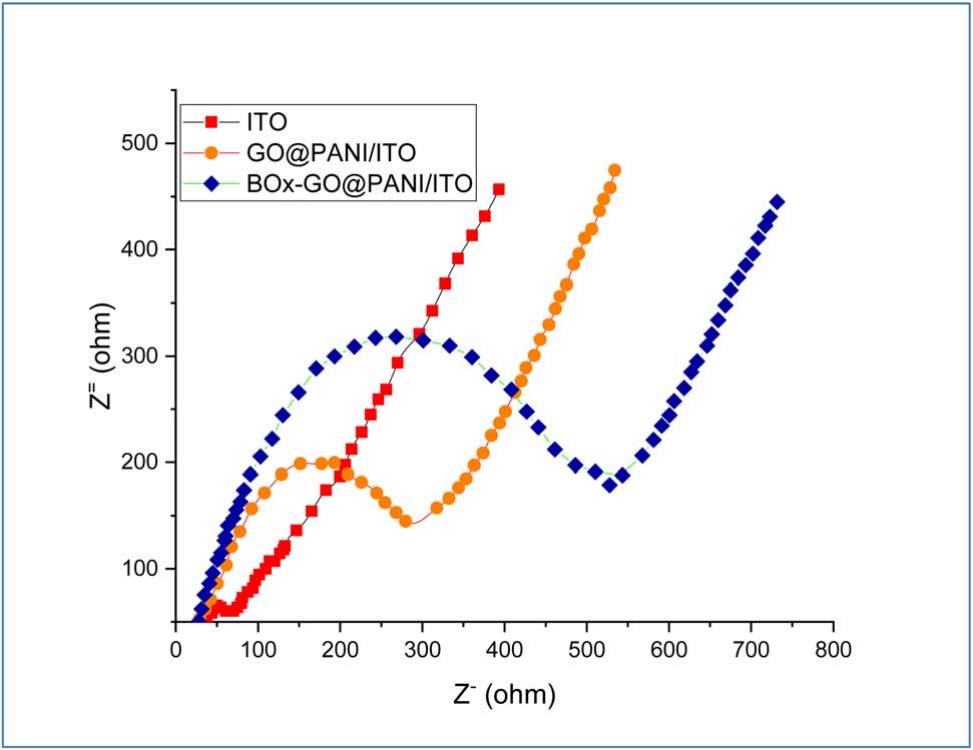
The Nyquist plots of fabricated electrodes.

To study the alteration in the electrode surface impedance, electrochemical impedance spectroscopy (EIS) and cyclic voltammetry measurements were carried out. This tool is sensitive to the sequential stages of the biosensor electrode fabrication, which emphasizes successful modification of the electrode. The EIS spectrum of the fabricated electrodes is shown in Fig. 7. The results showed two frequencies; a lower frequency corresponding to the Warburg diffusion and a high frequency assigned to the Nyquist plot which equaled the resistance of the charge transfer (*R*_ct_).^45,88^ The Nyquist plot was created by using the fabricating electrodes and electrolyte solution containing 0.1 M sodium phosphate buffer and 5 mM K_4_Fe(CN)_6_/K_3_Fe(CN)_6_ with ratio (1 : 1) as the redox probe. From the Nyquist plot, the results showed that *R*_ct_ values of bare ITO, GO@PANI/ITO, and BOx/GO@PANI/ITO electrodes were 730, 300, and 515 Ω, respectively, which indicate that the GO@PANI/ITO electrode has more conductivity, lower resistance, and more electron transfer efficiency than the bare ITO electrode. Moreover, incorporating GO nanosheets with PANI provides more capacitance and many active sites for faradaic reactions. On the other, the results demonstrated that the *R*_ct_ value of BOx/GO@PANI/ITO is higher than that of GO@PANI/ITO after immobilization of BOx, which may be back to the hydrophobic materials and high thickness that causes more electron transfer resistance.

**Fig. 8:**
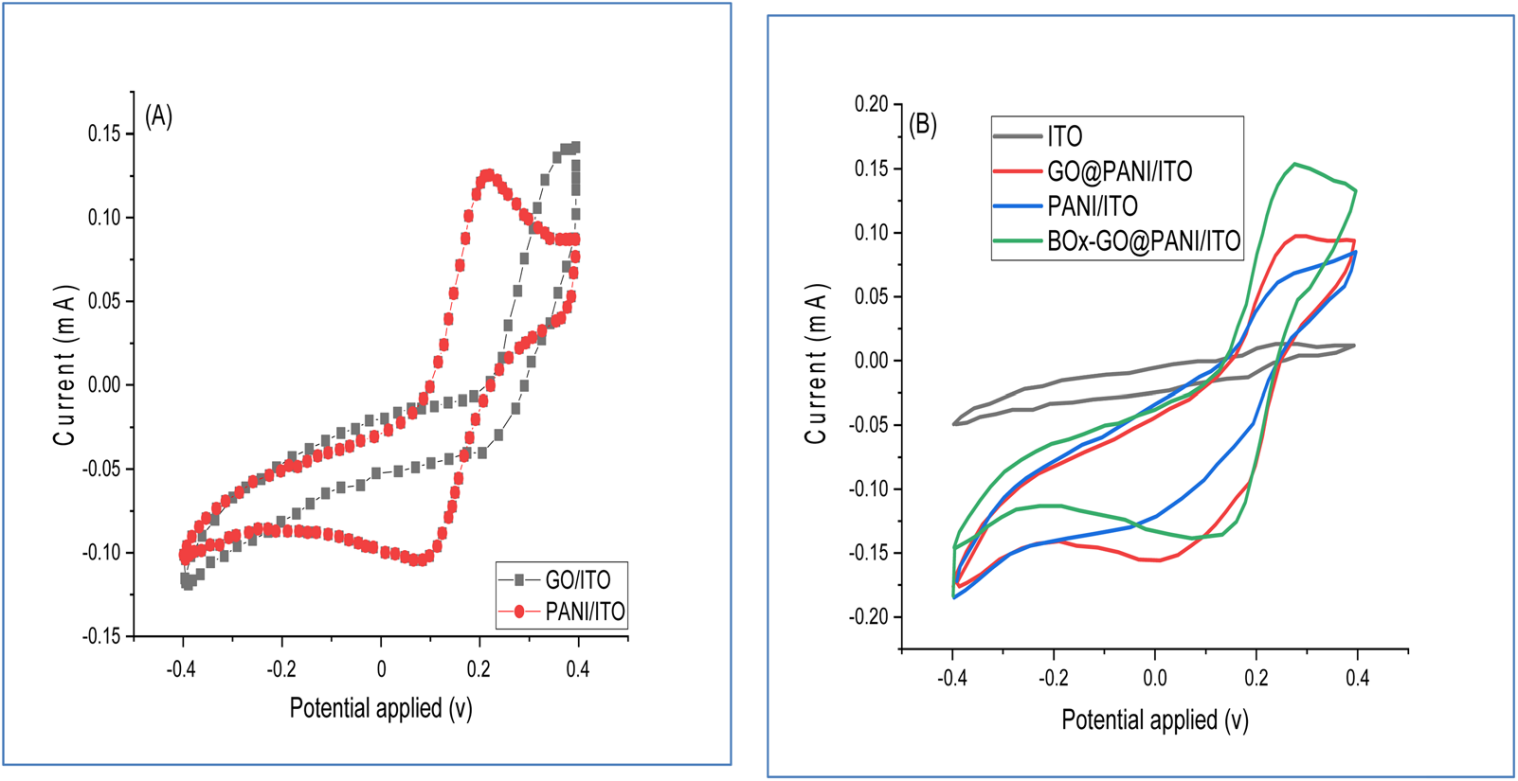
CV of (A) GO/ITO and PANI/ITO electrodes; (B) CV of the fabricated electrodes in sodium phosphate buffer at pH 7.5, 30 °C in 0.1 mM bilirubin.

By using cyclic voltammetry (CV) analysis, a comparison between GO, PANI, and GO-PANI-modified ITO electrodes was performed. The measurements were carried out using 0.1 M sodium phosphate buffer and 5 mM K_4_Fe(CN)_6_/K_3_Fe(CN)_6_ with a ratio (1 : 1) as an electrolyte solution, and using three electrodes GO, PANI, and GO-PANI modified ITO in separate experiments. The CVs were recorded in different scan rates with a potential window range of −0.4 to +0.4, as illustrated in Fig. 8A. The results showed that the current values of GO/ITO and PANI/ITO are 0.14 mA and 0.16 mA, respectively. The current response of CV was increased for the electrode modified by GO nanosheets as shown in curve *a*, in which the deposition of GO led to a faster increase in the intensity of the current as a result of increasing the active area of the electrode. Fig. 8B shows the consecutive fabrication of the enzyme electrode that was investigated using CV measurements at a potential equal to +0.2 V. The data did not show any redox peaks for bare ITO electrodes, as shown in Fig. 8B (black line), while it spotted the current characteristic for the PANI/ITO electrode after 10 scan rates (Fig. 8B/blue line). As shown in Fig. 8B (red line), the CVs of GO/PANI/ITO showed an increased level of current with good redox peaks, corresponding to the presence of GO nanosheets. At 0.15 mA, the BOx/GO/PANI/ITO electrode showed a redox peak assigned to H_2_O_2_ oxidation *via* immobilized BOx, as demonstrated in Fig. 8B (green line).

### 3.4 Bilirubin biosensor response

**Fig. 9:**
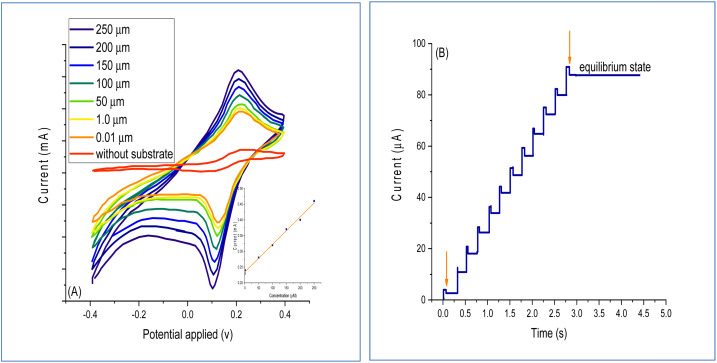
CV of (A) the BOx-GO@PANI/ITO electrode at different concentrations in the sodium phosphate buffer at pH 7.5 and a scan rate of 50 mV. (B) Chronoamperometric curves investigated at different concentrations of bilirubin.

The response investigation of the BOx/GO@PANI/ITO electrode in the concentration of bilirubin from 0.01 to 250 μm utilizing the phosphate buffer solution is illustrated in Fig. 9A. The results indicated a linear relationship, which is in agreement with earlier studies.^46^Fig. 9B shows the plots of the current–time for different bilirubin concentrations. The voltammetric calculations were performed after the addition of 250 μm of bilirubin concentration. A considerable increase in the current was not noted when the bilirubin concentration was increased beyond 250 μM, indicating that the fabricated BOx/GO@PANI/ITO electrode reached a saturation level at 250 μM. The results indicated that the time demanded to achieve 95% of the steady-state response was 2.5 s, which indicated a fast process. Moreover, the LOD and LOQ values of the fabricated sensor were calculated and the results showed that they were 0.15 and 2.8 nM, respectively. In addition to this, the fabricated biosensor alteration of quartz crystal is performed utilizing hydroxyapatite film during the molecular imprinting process using the sol–gel surface technique.^46^

### 3.5 Biosensor optimization

To examine the GO amount that can be precipitated with PANI, its concentration ranged from 0.1–10 mg ml^−1^ in the NaClO_4_ + aniline + GO nanosheets solution and the response of the chronoamperometric current was registered and summarized in Table 1. Although the anodic current increased with increasing concentration of GO nanosheets, following changes in the morphology of the GO/PANI nanocomposite, the essential improvements were not noted across the higher GO nanosheet concentrations. Then, a 5 mg ml^−1^ concentration was used for fabricating the working electrode.^47^ The electro polymerization mechanism of aniline marked by the oxidation of aniline monomer formed a solution rich in aniline cations at the surface of electrode. This unstable aniline cation may react with anions present in the solution to produce soluble products or may bond with GO nanosheets by van der Waals forces or hydrogen bonding.^48^ GO nanosheets have a high surface area, which provides multiple fixed sites for soluble products with low molecular weight. In all the experiments, the voltage value of +0.2 V was used as the standard, where the best response of the fabricated biosensor was noted. The impact of temperature, pH, bilirubin concentration, and incubation time were estimated as these factors impacted the conditions of the experiment in response to the fabricated biosensor. The results indicated that the optimum temperature and pH were 30 °C and 7.5, respectively. Compared to the reported studies,^48,89^ the optimum pH optima was lower than that of the controlled bilirubin based on the indirect electrochemical response. On the other hand, the results showed that the obtained relationship between the response of the biosensor and bilirubin concentration (0.01–250 μm) was linear, and the response remained constant after 250 μm. The fabricated biosensor displayed a high sensitivity of 0.905 μA μm^−1^. Moreover, the response of the biosensor was quick and was recorded as 2 s from 95% of the constant current for each point.

**Table 1:** The current response at different concentrations of the nanocomposite

Solution [NaClO_4_ (100 μM + 30 μM aniline)]	Concentration of GO Np (mg L^−1^)	Current (mA)
NaClO_4_ + aniline	0.1	0.17
	1	0.26
	2.5	0.29
	5	0.31
	10	0.32

### 3.6 Bilirubin detection in real samples

**Fig. 10:**
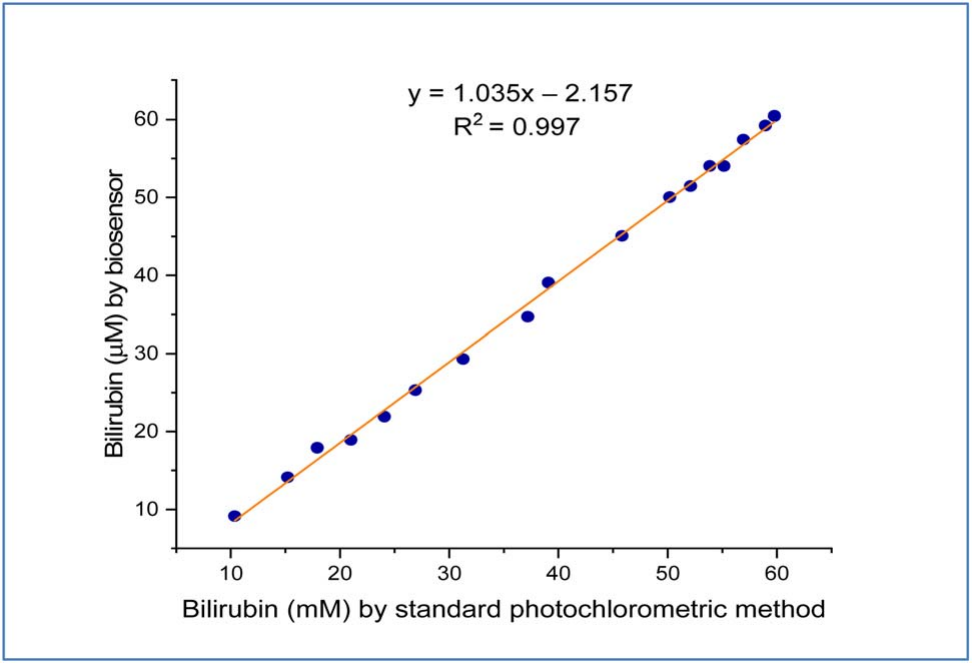
The relationship between the fabricated biosensor and that measured using a standard photochromatic method.

In the serum samples of healthy and patients with jaundice (ESI† Table. 1), the levels of bilirubin measured using the fabricated biosensor ranged from 0.2–15 and 20–60 μm, respectively. To estimate the precision of this method, twenty samples were contrasted for detecting bilirubin using the BOx/GO@PANI/ITO electrode (*y*) and the common colorimetric method (*x*). The results show that the correlation analysis displayed a linear relationship by utilizing a regression equation, with determination coefficient *R*^2^ equal to 0.997, and the equation of regression was *y* = 1.035*x* − 2.157, as shown in Fig. 10. These results show that the performance of the fabricated biosensor in serum samples showed a good response compared with another biosensor.

**Fig. 11:**
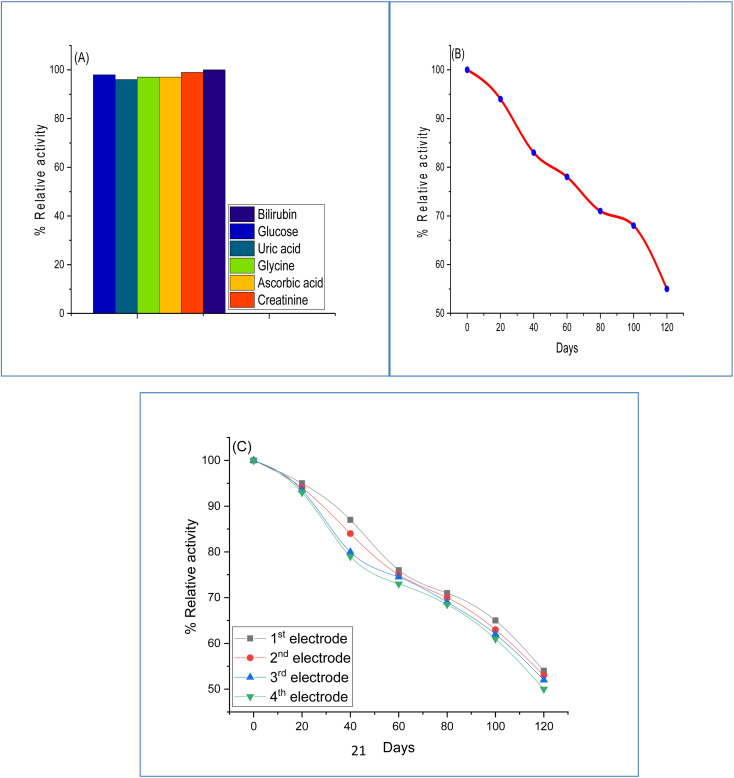
(A) The effect of the interferences on the activity of the fabricated biosensor, (B) response of the fabricated biosensor under 5 °C storage conditions, and (C) the effect of storage stability on four similar fabricated biosensors.

To compare the difference response of amperometry, many interferences, involving 5 mM of glucose, uric acid, glucine, ascorbic acid, and creatinine were added. For all the measurements, the constants were bilirubin conc. 100 μM, pH 7.5, and the sodium phosphate buffer solution. The results demonstrated that the activity of interference was reduced as follows: 2% glucose, 4% uric acid, 3% glycine and ascorbic acid, and 1% creatinine, as shown in Fig. 11A, which indicates that there was no impact on the practical impact on the response of the biosensor.

To investigate the stability of the fabricated biosensor with time, the current response was determined by storing the biosensor at 5 °C. The results (Fig. 11B) show that the fabricated enzyme electrode maintained 50% of the initial activity after using it 100 times for 120 days, illustrating considerable agreement with those reported earlier.^49^ Four enzyme electrodes were created and estimated individually for the effect of storage at 5 °C. The results (Fig. 11C) show that no considerable difference in the stability of storage of fabricated electrode was noted marking a reproducible and satisfactory performance showing high stability to a higher frequency for utilizing the fabricated electrode.

Compared with the anterior studies or analytical methods, for example, the piezoelectric and electrochemical methods (Table 2), this sensor showed good sensitivity, lower limit of detection, and faster achievement for the detection of bilirubin. The linear range of this enzyme biosensor BOx/GO@PANI/ITO for bilirubin was approximately 0.01 to 250 μm. The uniformity of the results in our method compared to the standard method was noted. The fabricated biosensor BOx/GO@PANI/ITO showed application for the point-of-care testing for rapid *in vitro* characterization of jaundice.

**Table 2:** A comparison of analytical properties of bilirubin biosensors

Electrochemical sensor type	Sensing modified electrodes	Limit of detection (μM)	Detection linear range (μM)	Response (s)	Storage stability (days)	Ref.
Amperometric	(SiO_2_@ZrONPs)/chitosan/Au	0.02	0.02–250	2	120	45
Amperometric	Ppy/PANI film	0.01	0.01–320	2	60	46
Piezoelectric	TiO2 film	0.05	0.1–50	1800	90	47
Electrochemical	Au/MWCNTs	0.1	1–100	5	60	48
Amperometric	PEI film	0.04	0.1–50	5	60	49
Amperometric	Screen printed electrodes functionalized with carbon nanotubes and graphene	0.0001	0.1–600	ND	28	90
Amperometric	Europium doped yttrium oxide	0.041	0.0–60	ND	ND	91
Piezoelectric	Paper-based screen-printed electrodes functionalized with silver nanoparticles	0.1	0.1–90	ND	ND	92
Electrochemical	Molecular imprinted polymer and ferromagnetic nanocomposite	0.15	0.03–0.13	ND	ND	93
Amperometric	BOx/GO@PANI/ITO	0.01	0.01–250	2	120	Our study

## 4. Conclusions

In this work, an enzyme biosensor that can measure the free type of bilirubin with good selectivity and sensitivity in serum was fabricated. The synthesized GO/PANI nanocomposite combined the high transfer ability of electrons, hence noting enhancement of the sensor performance. The utilization of BOx/GP@PANI modified ITO has expedited the biosensing of bilirubin providing analytical improvement from the limit of detection that is 0.1 nM and a wide range of 0.01–250 μM for working concentrations, fast response (3 s) and the stability of the storage for 100 days without any interference with materials.

## Author contributions

Noor Sabah Ahmed, Chou-Yi Hsu, Zaid H. Mahmoud, Hamidreza Sayadi, Ehsan Kianfar: Investigation, writing – original draft, reviewing, and editing. All authors have read and approved the final manuscript.

## Abbreviations

TGA: Thermogravimetric analysis
DTG: Derivative thermogravimetry
Glu: Glutaraldehyde

## Conflicts of interest

The authors declare that they have no competing interests.

## Supplementary Material

RA-013-D3RA06815C-s001

## References

[cit1] Athanassiadis S., Chopra D. R., Fischer M. A., Menna J. (1974). An electrophoretic method for detection of unbound bilirubin and reserve bilirubin binding capacity in serum of newborns. J. Lab. Clin. Med..

[cit2] Pan X., Li L., Lin H., Tan J., Wang H., Liao M., Chen C., Shan B., Chen Y., Li M. (2019). A graphene oxide-gold nanostar hybrid based-paper biosensor for label-free SERS detection of serum bilirubin for diagnosis of jaundice. Biosens. Bioelectron..

[cit3] Santhosh M., Chinnadayyala S. R., Kakoti A., Goswami P. (2014). Selective and sensitive detection of free bilirubin in blood serum using human serum albumin stabilized gold nanoclusters as fluorometric and colorimetric probe. Biosens. Bioelectron..

[cit4] Ellairaja S., Shenbagavalli K., Ponmariappan S., Vasantha V. S. (2017). A green and facile approach for synthesizing imine to develop optical biosensor for wide range detection of bilirubin in human biofluids. Biosens. Bioelectron..

[cit5] Olusanya B. O., Kaplan M., Hansen T. W. R. (2018). Neonatal hyperbilirubinaemia: a global perspective. Lancet Child Adolesc. Health.

[cit6] Kuang Z., Zong X., Xing S., Zhao F., Guo S., Li H., Wei D. (2021). Analytical performance validation and clinical application of blood gas analyzer on the detection of neonatal bilirubin. Transl. Pediatr..

[cit7] Guerra Ruiz A. R., Crespo J., López Martínez R. M., Iruzubieta P., Casals Mercadal G., Lalana Garcés M., Morales Ruiz M. (2021). Measurement and clinical usefulness of bilirubin in liver disease. Adv. Lab. Med..

[cit8] Iwatani S., Nakamura H., Kurokawa D. (2016). *et al.*, Fluorescent protein-based detection of unconjugated bilirubin in newborn serum. Sci. Rep..

[cit9] Anjana R. R., Anjali Devi J. S., Jayasree M. (2018). *et al.*, S,N-doped carbon dots as a fluorescent probe for bilirubin. Microchim. Acta.

[cit10] Mreihil K., Madsen P., Nakstad B. (2015). *et al.*, Early formation of bilirubin isomers during phototherapy for neonatal jaundice: effects of single *vs.* double fluorescent lamps *vs.* photodiodes. Pediatr. Res..

[cit11] Pinyorospathum C., Chaiyo S., Sae-ung P. (2019). *et al.*, Disposable paper-based electrochemical sensor using thiol-terminated poly(2-methacryloyloxyethyl phosphorylcholine) for the label-free detection of C-reactive protein. Microchim. Acta.

[cit12] George J. M., Antony A., Mathew B. (2018). Metal oxide nanoparticles in electrochemical sensing and biosensing: a review. Microchim. Acta.

[cit13] Wang C., Wang G., Fang B. (2009). Electrocatalytic oxidation of bilirubin at ferrocenecarboxamide modified MWCNT–gold nanocomposite electrodes. Microchim. Acta.

[cit14] Jamshidi M., Walcarius A., Thangamuthu M. (2023). *et al.*, Electrochemical approaches based on micro- and nanomaterials for diagnosing oxidative stress. Microchim. Acta.

[cit15] Anand S. K., Mathew M. R., Kumar K. G. (2020). A Simple and Cost Effective Turn off Fluorescence Sensor for Biliverdin and Bilirubin Based on l-Cysteine Modulated Copper Nanoclusters. J. Fluoresc..

[cit16] Boonkaew S., Chaiyo S., Jampasa S. (2019). *et al.*, An origami paper-based electrochemical immunoassay for the C-reactive protein using a screen-printed carbon electrode modified with graphene and gold nanoparticles. Microchim. Acta.

[cit17] Negahdary M., Behjati-Ardakani M., Heli H. (2019). An electrochemical troponin T aptasensor based on the use of a macroporous gold nanostructure. Microchim. Acta.

[cit18] Makizuka T., Sowa K., Shirai O. (2022). *et al.*, Inhibition of direct-electron-transfer-type bioelectrocatalysis of bilirubin oxidase by silver ions. Anal. Sci..

[cit19] Xiong L. Y., Kim Y. J., Seo W. C. (2023). *et al.*, High-performance non-enzymatic glucose sensor based on Co_3_O_4_/rGO nanohybrid. Rare Met..

[cit20] Yu H., Li R., Song Kl. (2019). Amperometric determination of nitrite by using a nanocomposite prepared from gold nanoparticles, reduced graphene oxide and multi-walled carbon nanotubes. Microchim. Acta.

[cit21] Yao J., Wang H., Chen M. (2019). *et al.*, Recent advances in graphene-based nanomaterials: properties, toxicity and applications in chemistry, biology and medicine. Microchim. Acta.

[cit22] Ali H., Verma N. (2022). A Cu–CNF–rGO-functionalized carbon film indicated as a versatile electrode for sensing of biomarkers using electropolymerized recognition elements. J. Mater. Sci..

[cit23] Raya I., Kzar H. H., Mahmoud Z. H. (2022). *et al.*, A review of gas sensors based on carbon nanomaterial. Carbon Lett..

[cit24] Mahdi M. A., Farhan M. A., Mahmoud Z. H., Rheima A. M., Abbas Z. sabri, Kadhim M. M., Jaber A. S., Hachim S. K., Ismail A. H. (2023). Direct sunlight photodegradation of congo red in aqueous solution by TiO_2_/rGO binary system: Experimental and DFT study. Arabian J. Chem..

[cit25] Hsu C. Y., Rheima A. M., Mohammed M. S. (2023). *et al.*, Application of Carbon Nanotubes and Graphene-Based Nanoadsorbents in Water Treatment. J. Bionanosci..

[cit26] AbdulKareem E. A., Mahmoud Z. H., Khadom A. A. (2023). Sunlight assisted photocatalytic mineralization of organic pollutants over rGO impregnated TiO_2_ nanocomposite: Theoretical and experimental study. Case Stud. Chem. Environ. Eng..

[cit27] Mahmoud Z. H., AL-Bayati R. A., Khadom A. A. (2022). Electron transport in dye-sanitized solar cell with tin-doped titanium dioxide as photoanode materials. J. Mater. Sci.: Mater. Electron..

[cit28] Bokov D. O., Mustafa Y. F., Mahmoud Z. H. (2022). *et al.*, Cr-SiNT, Mn-SiNT, Ti-C70 and Sc-CNT as Effective Catalysts for CO_2_ Reduction to CH_3_OH. Silicon.

[cit29] Mahmoud Z. H., Al-Bayati R. A., Khadom A. A. (2021). Enhanced photovoltaic performance of dye-sanitized solar cell with tin doped titanium dioxide as photoanode materials. Chalcogenide Lett..

[cit30] Abdul-Reda Hussein U., Mahmoud Z. H., Abd Alaziz K. M., Alid M. L., Yasin Y., Ali F. K., An F., An A., Kianfar E. (2023). Antimicrobial finishing of textiles using nanomaterials. Braz. J. Biol..

[cit31] Mahmoud Z. H., AL-Bayati R. A., Khadom A. A. (2022). Synthesis and supercapacitor performance of polyaniline-titanium dioxide-samarium oxide (PANI/TiO2-Sm2O3) nanocomposite. Chem. Pap..

[cit32] Mahmoud Z. H., Al-Bayati R. A., Khadom A. A. (2022). *In situ* Polymerization of Polyaniline/Samarium Oxide – Anatase Titanium Dioxide (PANI/Sm_2_O_3_-TiO2) Nanocomposite: Structure. Thermal and Dielectric Constant Supercapacitor Application Study.

[cit33] Al-Obaidi N. S., Mahmoud Z. H., Ali A. A. F. A. S., Ali F. K. (2018). Evaluating the electric properties of poly aniline with doping ZnO and α-Fe_2_O_3_ nanoparticles. Pharmacophore.

[cit34] Woronyczová J., Nováková M., Leníček M. (2022). *et al.*, Serum Bilirubin Concentrations and the Prevalence of Gilbert Syndrome in Elite Athletes. Sports Med..

[cit35] Groshkova Y. A., Buslaeva E. Y., Gubin S. P. (2019). Transformation of graphene oxide in supercritical media. Russ. Chem. Bull..

[cit36] Zhang F., Wang B., He S., Man R. (2014). Preparation of graphene-oxide/polyamidoamine dendrimers and their adsorption properties toward some heavy metal ions. J. Chem. Eng. Data.

[cit37] Zhang W. L., Park B. J., Choi H. J. (2010). Colloidal graphene oxide/polyaniline nanocomposite and its electrorheology. Chem. Commun..

[cit38] Athawale A. A., Kulkarni M. V., Chabukswar V. V. (2002). Studies on chemically synthesized soluble acrylic acid doped polyaniline. Mater. Chem. Phys..

[cit39] Mitra M., Kulsi C., Chatterjee K., Kargupta K., Ganguly S., Banerjee D. (2015). *et al.*, Reduced graphene oxide-polyaniline composites—synthesis, characterization and optimization for thermoelectric applications. RSC Adv..

[cit40] Pimenta M. A., Dresselhaus G., Dresselhaus M. S., Cançado L. G., Jorio A., Saito R. (2007). Studying disorder in graphite-based systems by Raman spectroscopy. Phys. Chem. Chem. Phys..

[cit41] Ahmad N., Karim S., Hussain D. (2022). *et al.*, Efficient dual adsorption of eosin Y and methylene blue from aqueous solution using nanocomposite of graphene oxide nanosheets and ZnO nanospheres. Korean J. Chem. Eng..

[cit42] Zhang S., Chen S., Cao Y. (2019). *et al.*, Polyaniline nanoparticle coated graphene oxide composite nanoflakes for bifunctional multicolor electrochromic and supercapacitor applications. J. Mater. Sci.: Mater. Electron..

[cit43] Kou L., Gao C. (2011). Making silicananoparticle-covered graphene oxide nanohybrids as general building blocks for large-area superhydrophilic coatings. Nanoscale.

[cit44] Lee C. Y., Bae J.-H., Kim T.-Y., Chang S.-H., Kim S. Y. (2015). Using silane-functionalized graphene oxides for enhancing the interfacial bonding strength of carbon/epoxy composites. Composites, Part A.

[cit45] Male U., Srinivasan P., Singu B. S. (2015). Incorporation of polyaniline nanofibres on graphene oxide by interfacial polymerization pathway for supercapacitor. Int. Nano Lett..

[cit46] Santhosh M., Chinnadayyala S. R., Kakoti A., Goswami P. (2014). Selective and sensitive detection of free bilirubin in blood serum using human serum albumin stabilized gold nanoclusters as fluorometric and colorimetric probe. Biosens. Bioelectron..

[cit47] Sadki S., Schottland P., Brodie N., Sabouraud G. (2000). The mechanisms of pyrrole electropolymerization. Chem. Soc. Rev..

[cit48] Wang C., Wang G., Fang B. (2009). Electrocatalytic oxidation of bilirubin at ferrocenecarboxamide modified MWCNT–gold nanocomposite electrodes. Microchim. Acta.

[cit49] Pita M., Gutierrez-Sanchez C., Toscano M. D., Shleev S., De Lacey A. L. (2013). Oxygen biosensor based on bilirubin oxidase immobilized on a nanostructured gold electrode. Bioelectrochemistry.

[cit50] AL-Salman H. N. K., sabbar Falih M., Deab H. B., Altimari U. S., Shakier H. G., Dawood A. H., Kianfar E. (2023). A study in analytical chemistry of adsorption of heavy metal ions using chitosan/graphene nanocomposites. Case Stud. Chem. Environ. Eng..

[cit51] pour G. B., Shajee nia E., Darabi E., Fekri aval L., Nazarpour-Fard H., Kianfar E. (2023). Fast NO2 gas pollutant removal using CNTs/TiO2/CuO/zeolite nanocomposites at the room temperature. Case Stud. Chem. Environ. Eng..

[cit52] Hsu C. Y., Rheima A. M., Kadhim M. M., Ahmed N. N., Mohammed S. H., Abbas F. H., Kianfar E. (2023). An overview of nanoparticles in drug delivery: properties and applications. S. Afr. J. Chem. Eng..

[cit53] Hsu C. Y., Rheima A. M., Mohammed M. S., Kadhim M. M., Mohammed S. H., Abbas F. H., Mahmoud Z. H. (2023). Application of Carbon Nanotubes and Graphene-Based Nanoadsorbents in Water Treatment. J. Bionanosci..

[cit54] sabri Abbas Z., Kadhim M. M., Mahdi Rheima A., jawad al-bayati A. D., Talib Abed Z., dashoor Al-Jaafari F. M., Kianfar E. (2023). Preparing Hybrid Nanocomposites on the Basis of Resole/Graphene/Carbon Fibers for Investigating Mechanical and Thermal Properties. J. Bionanosci..

[cit55] Hsu C. Y., Abdullaev Z. H. M. S., Mohammed B. A., Altimari U. S., Shaghnab M. L., kianfar E., Smaisim G. F. (2023). Nanocomposites based on Resole/graphene/carbon fibers: a review study. Case Stud. Chem. Environ. Eng..

[cit56] Smaisim G. F., Abed A. M., Al-Madhhachi H., Hadrawi S. K., Al-Khateeb H. M. M., Kianfar E. (2023). Graphene-based important carbon structures and nanomaterials for energy storage applications as chemical capacitors and supercapacitor electrodes: a review. J. Bionanosci..

[cit57] Kadhim M. M., Rheima A. M., Abbas Z. S., Jlood H. H., Hachim S. K., Kadhum W. R., kianfar E. (2023). Evaluation of a biosensor-based graphene oxide-DNA nanohybrid for lung cancer. RSC Adv..

[cit58] Hachem K., Ansari M. J., Saleh R. O., Kzar H. H., Al-Gazally M. E., Altimari U. S., Kianfar E. (2022). Methods of chemical synthesis in the synthesis of nanomaterial and nanoparticles by the chemical deposition method: A review. J. Bionanosci..

[cit59] Salahdin O. D., Sayadi H., Solanki R., Parra R. M. R., Al-Thamir M., Jalil A. T., Kianfar E. (2022). Graphene and carbon structures and nanomaterials for energy storage. Appl. Phys. A: Mater. Sci. Process..

[cit60] Abdelbasset W. K., Jasim S. A., Bokov D. O., Oleneva M. S., Islamov A., Hammid A. T., Kianfar E. (2022). Comparison and evaluation of the performance of graphene-based biosensors. Carbon Letters.

[cit61] Ansari M. J., Kadhim M. M., Hussein B. A., Lafta H. A., Kianfar E. (2022). Synthesis and stability of magnetic nanoparticles. J. Bionanosci..

[cit62] Bokov D., Turki Jalil A., Chupradit S., Suksatan W., Javed Ansari M., Shewael I. H., Kianfar E. (2021). Nanomaterial by sol-gel method: synthesis and application. Adv. Mater. Sci. Eng..

[cit63] Kianfar E. (2021). Magnetic nanoparticles in targeted drug delivery: a review. J. Supercond. Novel Magn..

[cit64] Kianfar E. (2021). Protein nanoparticles in drug delivery: animal protein, plant proteins and protein cages, albumin nanoparticles. J. Nanobiotechnol..

[cit65] Huang X., Zhu Y., Kianfar E. (2021). Nano biosensors: properties, applications and electrochemical techniques. J. Mater. Res. Technol..

[cit66] Kianfar E., Cao V. (2021). Polymeric membranes on base of PolyMethyl methacrylate for air separation: a review. J. Mater. Res. Technol..

[cit67] Raya I., Kzar H. H., Mahmoud Z. H., Al Ayub Ahmed A., Ibatova A. Z., Kianfar E. (2021). A review of gas sensors based on carbon nanomaterial. Carbon Letters.

[cit68] Gao C., Liao J., Lu J., Ma J., Kianfar E. (2021). The effect of nanoparticles on gas permeability with polyimide membranes and network hybrid membranes: a review. Rev. Inorg. Chem..

[cit69] mousavian S., Faravar P., Zarei Z., azimikia R., Monjezi M. G., kianfar E. (2020). Modeling and simulation absorption of CO2 using hollow fiber membranes (HFM) with mono-ethanol amine with computational fluid dynamics. J. Environ. Chem. Eng..

[cit70] Kianfar F., Kianfar E. (2019). Synthesis of isophthalic acid/aluminum nitrate thin film nanocomposite membrane for hard water softening. J. Inorg. Organomet. Polym. Mater..

[cit71] Kianfar E., Salimi M., Kianfar F., Kianfar M., Razavikia S. A. H. (2019). CO2/N2 separation using polyvinyl chloride iso-phthalic acid/aluminium nitrate nanocomposite membrane. Macromol. Res..

[cit72] Salimi M., Pirouzfar V., Kianfar E. (2017). Novel nanocomposite membranes prepared with PVC/ABS and silica nanoparticles for C_2_H_6_/CH_4_ separation. Polym. Sci., Ser. A.

[cit73] Salimi M., Pirouzfar V., Kianfar E. (2017). Enhanced gas transport properties in silica nanoparticle filler-polystyrene nanocomposite membranes. Colloid Polym. Sci..

[cit74] Alabada R., Kadhim M. M., sabri Abbas Z., Rheima A. M., Altimari U. S., Dawood A. H., Kianfar E. (2023). Investigation of Effective Parameters in the Production of Alumina Gel through the Sol–Gel Method. Case Stud. Chem. Environ. Eng..

[cit75] Rheima A. M., sabri Abbas Z., Kadhim M. M., Mohammed S. H., Alhameedi D. Y., Rasen F. A., Kianfar E. (2023). Aluminum oxide nano porous: Synthesis, properties, and applications. Case Stud. Chem. Environ. Eng..

[cit76] Al-Awsi G. R. L., Alameri A. A., Al-Dhalimy A. M. B., Gabr G. A., Kianfar E. (2023). Application of nano-antibiotics in the diagnosis and treatment of infectious diseases. Braz. J. Biol..

[cit77] Abdul-Reda Hussein U., Mahmoud Z. H., Abd Alaziz K. M., Alid M. L., Yasin Y., Ali F. K., Kianfar E. (2023). Antimicrobial finishing of textiles using nanomaterials. Braz. J. Biol..

[cit78] Younus L. A., Mahmoud Z. H., Hamza A. A., Alaziz K. M. A., Ali M. L., Yasin Y., Kianfar E. (2023). Photodynamic therapy in cancer treatment: properties and applications in nanoparticles. Braz. J. Biol..

[cit79] Li T., Pang H., Wu Q., Huang M., Xu J., Zheng L., Qiao Y. (2022). Rigid Schiff Base Complex Supermolecular Aggregates as a High-Performance pH Probe: Study on the Enhancement of the Aggregation-Caused Quenching (ACQ) Effect *via* the Substitution of Halogen Atoms. Int. J. Mol. Sci..

[cit80] Han S., Chen C., Chen C., Wu L., Wu X., Lu C., Hou J. (2023). Coupling annealed silver nanoparticles with a porous silicon Bragg mirror SERS substrate and machine learning for rapid non-invasive disease diagnosis. Anal. Chim. Acta.

[cit81] Chen X., Lv S., Kang J., Wang Z., Guo T., Wang Y., Guo L. (2023). Efficient C–N coupling in the direct synthesis of urea from CO_2_ and N_2_ by amorphous Sb_*x*_Bi_1−*x*_O_*y*_ clusters. Proc. Natl. Acad. Sci. U. S. A..

[cit82] Zhang J., Wang L., Zhong A., Huang G., Wu F., Li D., Han D. (2019). Deep red PhOLED from dimeric salophen Platinum(II) complexes. Dyes Pigm..

[cit83] Ma Y., Leng Y., Huo D., Zhao D., Zheng J., Yang H., Hou C. (2023). A sensitive enzyme-free electrochemical sensor based on a rod-shaped bimetallic MOF anchored on graphene oxide nanosheets for determination of glucose in huangshui. Anal. Methods.

[cit84] Tang T., Zhou M., Lv J., Cheng H., Wang H., Qin D., Liu X. (2022). Sensitive and selective electrochemical determination of uric acid in urine based on ultrasmall iron oxide nanoparticles decorated urchin-like nitrogen-doped carbon. Colloids Surf., B.

[cit85] Xiao Y., Gong W., Zhao M., Zhang M., Lu N. (2023). Surface-engineered prussian blue nanozymes as artificial receptors for universal pattern recognition of metal ions and proteins. Sens. Actuators, B.

[cit86] Li M., Guo Q., Chen L., Li L., Hou H., Zhao Y. (2022). Microstructure and properties of graphene nanoplatelets reinforced AZ91D matrix composites prepared by electromagnetic stirring casting. J. Mater. Res. Technol..

[cit87] Xu P., Yuan Q., Ji W., Yu R., Wang F., Huo N. (2022). Study on the annealing phase transformation mechanism and electrochemical properties of carbon submicron fibers loaded with cobalt. Mater. Express.

[cit88] Kong M., Yang M., Li R., Long Z., Zhang Y. Z., Huang J., Cui X., Zhang X., Said Y. B., Li Z., Li C. H. (2023). Graphene-based flexible wearable sensors: mechanisms, challenges, and future directions. Int. J. Adv. Manuf. Technol..

[cit89] Huang Z., Li C., Zhou Z., Liu B., Zhang Y., Yang M., Gao T., Liu M., Zhang N., Sharma S., Dambatta Y. S., Li Y. (2023). Magnetic Bearing: Structure, Model and Control strategy. Int. J. Adv. Des. Manuf. Technol..

[cit90] Thangamuthu M., Gabriel W. E., Santschi C., Martin O. J. F. (2018). Electrochemical sensor for bilirubin detection using screen printed electrodes functionalized with carbon nanotubes and graphene. Sensors.

[cit91] Yang W., Xia J., Zhou G., Jiang D., Li Q. (2018). Sensitive detection of free bilirubin in blood serum using b-diketone modified europium-doped yttrium oxide nanosheets as a luminescent sensor. RSC Adv..

[cit92] Anzar N., Suleman S., Kumar R., Rawal R., Pundir C. S., Pilloton R., Narang J. (2022). Electrochemical Sensor for Bilirubin Detection Using Paper-Based Screen-Printed Electrodes Functionalized with Silver Nanoparticles. Micromachines.

[cit93] Parnianchi F., Kashanian S., Nazari M., Peacock M., Omidfar K., Varmira K. (2022). Ultrasensitive electrochemical sensor based on molecular imprinted polymer and ferromagnetic nanocomposite for bilirubin analysis in the saliva and serum of newborns. Microchem. J..

