# Supplementary material for: A graphene oxide/polyaniline nanocomposite biosensor: synthesis, characterization, and electrochemical detection of bilirubin
Source: RSC Adv. 2023 Dec 12;13(51):36280–92. doi: 10.1039/d3ra06815c (PMC10714673; doi:10.1039/d3ra06815c)
Supplement: RA-013-D3RA06815C-s001 [file RA-013-D3RA06815C-s001.pdf]

Electronic Supplementary Material (ESI) for RSC Advances.
This journal is © The Royal Society of Chemistry 2023

Sup. Table. 1: Serum bilirubin levels in apparently healthy persons and jaundice patients, as measured by bilirubin biosensor based on BOx/GONP@PANI/ITO electrode

| sex | Age | Healthy person | sex | Age | Healthy person |
|---|---|---|---|---|---|
| M | 29 | 10±0.03 | F | 36 | 22±0.01 |
| M | 26 | 12±0.05 | F | 55 | 21±0.03 |
| F | 55 | 13±0.02 | M | 64 | 31±0.04 |
| M | 79 | 13±0.04 | F | 39 | 24±0.01 |
| F | 44 | 09±0.02 | M | 53 | 25±0.04 |
| F | 52 | 11±0.02 | M | 50 | 31±0.02 |
| M | 47 | 14±0.03 | M | 61 | 29±0.04 |
| F | 36 | 15±0.03 | F | 40 | 38±0.03 |
| F | 19 | 07±0.05 | F | 45 | 40±0.01 |
| M | 25 | 06±0.05 | F | 39 | 38±0.05 |
| F | 16 | 0.3±0.01 | M | 42 | 38±0.01 |
| F | 62 | 12±0.03 | M | 50 | 42±0.03 |
| M | 53 | 05±0.02 | F | 47 | 38±0.02 |
| M | 47 | 08±0.02 | M | 41 | 39±0.03 |
| F | 55 | 13±0.03 | M | 34 | 43±0.02 |
| M | 21 | 0.3±0.02 | M | 33 | 48±0.02 |
| M | 24 | 09±0.01 | F | 46 | 45±0.01 |
| F | 52 | 08±0.05 | M | 46 | 58±0.04 |
| M | 60 | 06±0.02 | F | 40 | 57±0.03 |
| F | 22 | 12±0.01 | F | 27 | 53±0.01 |
| M | 55 | 06±0.03 | F | 33 | 55±0.03 |
| M | 54 | 08±0.03 | M | 45 | 58±0.05 |
| F | 56 | 05±0.05 | F | 43 | 54±0.06 |
| F | 19 | 0.3±0.02 | F | 19 | 51±0.01 |
| M | 21 | 02±0.01 | M | 45 | 61±0.03 |
| M | 27 | 01±0.03 | F | 35 | 60±0.02 |
